# Detection and Characterization of Early Gastric Cancer

**DOI:** 10.3389/fonc.2022.855216

**Published:** 2022-07-22

**Authors:** Carlos Noronha Ferreira, Juliana Serrazina, Rui Tato Marinho

**Affiliations:** ^1^ Serviço de Gastrenterologia e Hepatologia, Hospital de Santa Maria, Centro Hospitalar Universitário Lisboa Norte, Lisbon, Portugal; ^2^ Clínica Universitária de Gastrenterologia, Faculdade de Medicina, Universidade de Lisboa, Lisbon, Portugal

**Keywords:** early gastric cancer, risk stratification, quality indicators, upper gastrointestinal endoscopy, advanced endoscopic imaging

## Abstract

In this review, we would like to focus on risk stratification and quality indicators of diagnostic upper gastrointestinal endoscopy in the detection and characterization of early gastric cancer. Preparation of the upper gastrointestinal tract with mucolytic agents or simethicone is often overlooked in the west, and this inexpensive step prior to endoscopy can greatly improve the quality of imaging of the upper digestive tract. Risk stratification based on epidemiological features including family history, *Helicobacter pylori* infection status, and tobacco smoking is often overlooked but may be useful to identify a subgroup of patients at higher risk of developing gastric cancer. Quality indicators of diagnostic upper gastrointestinal endoscopy are now well defined and include: minimal inspection time of 3 min, adequate photographic documentation of upper gastrointestinal landmarks, utilization of advanced endoscopic imaging technology including narrow band imaging and blue laser imaging to detect intestinal metaplasia and characterize early gastric cancer; and standardized biopsy protocols allow for histological evaluation of gastric mucosa and detection of atrophic gastritis and intestinal metaplasia. Finally, endoscopic and histologic classifications such as the Kimura–Takemoto Classification of atrophic gastritis and the OLGA–OLGIM classifications may help stratify patients at a higher risk of developing early gastric cancer.

## Introduction

Early gastric cancer (EGC) is defined as cancer occurring in the gastric mucosa and confined to the mucosa or submucosa, irrespective of lymph node metastasis (1). Patients with gastric mucosal atrophy and/or intestinal metaplasia affecting the gastric corpus alone and/or the antrum are at higher risk of gastric adenocarcinoma (2–4). Gastric cancer is the fifth most frequently diagnosed cancer and the third most common cause of death due to cancer, with the highest incidence being reported in Korea, Japan, and Mongolia (5). Population-based gastric cancer screening endoscopy programs in Japan for adults aged >50 years and in Korea for adults aged >40 years have resulted in the early detection of gastric cancer with a resultant significant decrease in mortality (5, 6). Image enhanced endoscopic technology (IEE), also called advanced endoscopic imaging, which includes Narrow Band Imaging (NBI), Flexible Spectral Imaging Color Enhancement (FICE), blue laser imaging (BLI), probe based Confocal Laser Endomicroscopy (pCLE), improves detection of gastric intestinal metaplasia, dysplasia, and early gastric cancer (2, 7, 8).

## Pre-endoscopic Stratification of Risk for Early Gastric Cancer

Although the optimal method for risk stratification of gastric cancer is still unclear, pre-endoscopic assessment of epidemiologic factors can help stratify patients at risk of gastric cancer (1). Gastric cancer is a multifactorial disease and risk factors for gastric cancer identified include older age, male gender, *Helicobacter pylori* (*H. pylori*) infection, and smoking (4–6, 9–13).

A family history of gastric cancer in a first-degree relative is significant as these patients have an approximately threefold greater risk of gastric cancer (9, 12). Concerning the location of gastric cancer, risk factors for cancer at the cardia include obesity and gastroesophageal reflux disease (5, 6, 9). For non-cardia gastric cancer, *H. pylori* infection and smoking have been identified as risk factors (5, 9). There is a predominance of non-cardia gastric cancer in poorly developed regions of the world, while cancer of the cardia is more frequent in highly developed countries, except China and Japan (5).

Due to the higher risk of gastric atrophy and cancer in patients with low serum pepsinogen I levels, serum pepsinogen I/II ratio <3 and positive *H. pylori* antibody titers, screening endoscopy is recommended in these patients (1, 2). However, in patients with severe gastric mucosal atrophy and previous infection with *H. pylori*, false negative results may be observed (1). Additionally, in countries with a high prevalence of *H. pylori* infection (>50%), the utility of serum pepsinogen levels has been questioned and considered irrelevant (1). Machine learning models involving the age of the patient, presence of intestinal metaplasia, and gastric ulcer predict a higher risk of developing gastric cancer after *H. pylori* eradication (14).

## Preparation of the Upper Digestive Tract Before Endoscopy

A high-quality examination during an upper gastrointestinal endoscopy requires optimal mucosal visualization (1, 2, 15). The use of mucolytic and defoaming agents, such as simethicone and N-acetylcysteine, improves the visibility of gastric mucosa compared to water and is associated with an increased rate of detection of early gastric cancer (15–19). Recommended in Japanese guidelines on endoscopic diagnosis of EGC but often overlooked in the west, the preparation of the upper gastrointestinal tract with a mucolytic agent 10 to 30 min before endoscopy is an inexpensive method, with a low frequency of adverse reactions/minimal patient burden, that can help obtain the optimal gastric cleanliness (1, 15, 20, 21). The quality of mucosal visualization should be recorded in the endoscopic report (15). Gastric peristalsis inhibitor drugs, namely, butilscopolamine and glucagon, should be considered where there is intense peristalsis to facilitate careful inspection of the gastric mucosa (1). The use of sedation and analgesia is recommended in anxious patients while performing screening endoscopy to improve the quality of endoscopic evaluation (1).

## Quality Indicators of Diagnostic Upper Gastrointestinal Endoscopy

Quality indicators of diagnostic upper gastrointestinal endoscopy are now well defined and include minimal inspection time of the stomach of at least 3 min, adequate photographic documentation of upper gastrointestinal landmarks, usage of image enhanced endoscopic (IEE) technology including narrow band imaging and blue laser imaging to detect intestinal metaplasia and characterize EGC, and standardized biopsy protocols with separate biopsies from the gastric antrum and corpus allow for histological evaluation of gastric mucosa and detection of atrophic gastritis and intestinal metaplasia (1, 2, 15, 22).

Adequate inspection time of the stomach significantly improves gastric cancer detection (1, 15). In a study involving 30,506 upper GI endoscopies in asymptomatic patients screened for gastric cancer, an observation time of more than 3 min significantly increased neoplasm detection rates (13). Identification of a gastric mucosa with a high risk of early gastric cancer is important (23). The presence of gastric atrophy and intestinal metaplasia, thickened mucosal folds in the gastric corpus and xanthoma are associated with a higher risk of gastric cancer (1, 24).

Systematic evaluation of the stomach with photographic documentation is highly recommended by the Japanese and European Gastrointestinal Endoscopy Societies (1, 2, 22). A systematic screening protocol for the stomach allows for adequate mapping (22, 23).

High definition endoscopy with chromoendoscopy (CE) is better than high definition white-light endoscopy alone for the diagnosis of intestinal metaplasia and gastric dysplasia and EGC (25). Virtual CE, with or without magnification, should be used for the diagnosis of gastric precancerous conditions, for guiding biopsies for staging atrophic and metaplastic changes, and helping target neoplastic lesions (2, 26). IEE technology, including NBI and Blue Laser imaging (BLI), has been shown to be as useful as conventional chromoendoscopy with indigo carmine in characterizing early gastric cancer (1, 2, 22).

## Endoscopic and Histologic Classifications of Gastric Atrophy and Intestinal Metaplasia and Risk of Gastric Cancer

Endoscopic and histologic classifications such as the Kimura–Takemoto Classification of atrophic gastritis and the Operative Link on Gastritis Assessment (OLGA) and Operative Link on Gastric Intestinal Metaplasia assessment (OLGIM) classifications (**Table 1**) may help stratify patients at higher risk of developing early gastric cancer (1, 2, 27–29). The Kyoto classification, which includes 5 endoscopic findings: gastric atrophy, intestinal metaplasia, thickened gastric folds, mucosal nodularity, diffuse redness, and the presence or absence of regular arrangement of collecting venules, has been shown to be associated with *H. pylori* infection and gastric cancer risk (24).

**Table 1:** Operative link on gastric atrophy and gastric intestinal metaplasia assessment classification.

A.					
Atrophy score		Corpus			
		No atrophy (Score 0)	Mild atrophy (Score 1)	Moderate atrophy (Score 2)	Severe atrophy (Score 3)
**Antrum (Including incisura angularis)**	**No atrophy (Score 0)**	Stage 0	Stage I	Stage II	Stage II
	**Mild atrophy (Score 1)**	Stage I	Stage I	Stage II	Stage III
	**Moderate atrophy (Score 2)**	Stage II	Stage II	Stage III	Stage IV
	**Severe atrophy (Score 3)**	Stage III	Stage III	Stage IV	Stage IV
B.					
IM score		Corpus			
		No IM (Score 0)	Mild IM (Score 1)	Moderate IM (Score 2)	Severe IM (Score 3)
**Antrum (Including incisura angularis)**	**No IM (Score 0)**	Stage 0	Stage I	Stage II	Stage II
	**Mild IM (Score 1)**	Stage I	Stage I	Stage II	Stage III
	**Moderate IM (Score 2)**	Stage II	Stage II	Stage III	Stage IV
	**Severe IM (Score 3)**	Stage III	Stage III	Stage IV	Stage IV

Operative link on gastritis assessment staging system **(A)** and operative link on gastric intestinal metaplasia assessment **(B)** staging system. IM, intestinal metaplasia; OLGA, Operative link on gastritis assessment system; OLGIM, Operative link on gastric intestinal metaplasia assessment.

Adapted from Weng CY et al. (27) Higher intensity of colour means higher risk of Early Gastric Cancer.

Endoscopic grading of gastric intestinal metaplasia (EGGIM) score has shown excellent correlation with the OLGIM classification and is determined by the presence of intestinal metaplasia detected by image enhanced endoscopy by narrow band imaging or blue laser imaging which detects light blue crest (LBC), white opaque substance or tubulovillous mucosal pattern in each of the five areas (lesser and greater curvatures of the gastric antrum and corpus and incisura) and is scored as 0 (none), 1 (focal, ≤30%), or 2 (extensive, >30%) (25, 26, 30).

**Figure 1:**
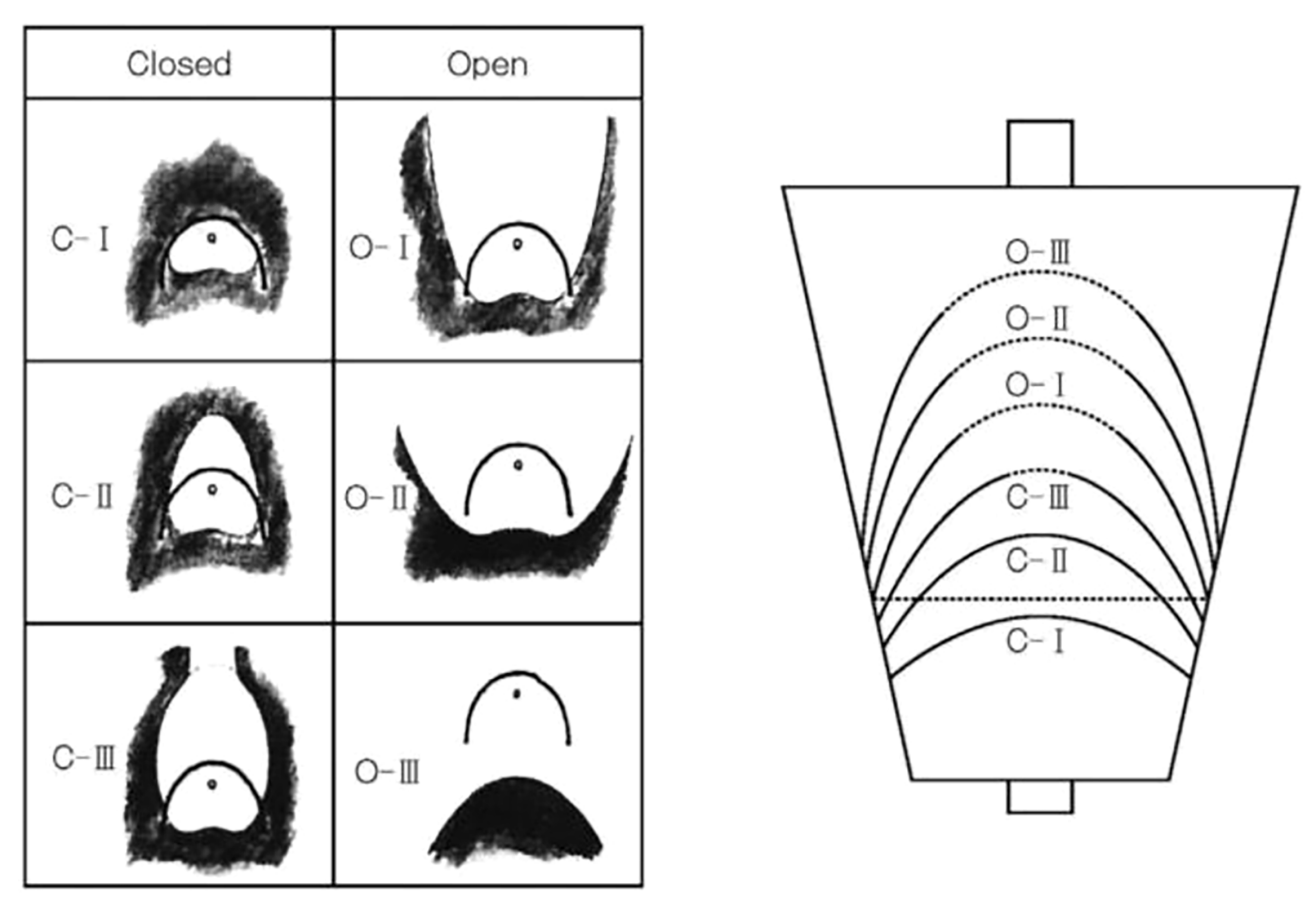
Kimura-Takemoto classification of endoscopic gastric mucosal atrophy. Reproduced with permission of the publishers from (1)

As shown in **Figure 1**, the Kimura–Takemoto Classification divides gastritis into open (O) and closed (C) types with three subdivisions in each of these main subtypes (O1–3, C1–3). In the closed type, atrophic mucosa is limited to: the antrum in C-1; the incisura angularis or the lower corpus and antrum in C2; the upper corpus extending to the cardia and involving the antrum in C3. In the open type, atrophic mucosa extends to the fundus over the cardia and the atrophic border of the body lies between the lesser curvature and the anterior wall with maintained folds of the greater curvature in O1; O2 is an intermediate type between O1 and O3, extending to the anterior and posterior walls of the corpus but not involving the greater curvature with the atrophic border on the anterior wall of the stomach; and in O3, atrophy is present in the entire stomach, with a lack of folds in the greater curvature as a whole (3).

In a study involving 27,777 patients, the prevalence of gastric cancer was 0% (0/4,506) for C1, 0.25% (9/3,660) for C2, 0.71% (21/2,960) for C3, 1.32% (75/5,684) for O1, 3.70% (140/3,780) for OII, and 5.33% (160/3,004) for O3 (3). In another study involving 573 patients with gastritis, after eradication of *H. pylori*, the cumulative 5-year incidence of gastric cancer was 1.5% in those without intestinal metaplasia, 5.3% in those with intestinal metaplasia in the gastric antrum, and 9.8% in those with intestinal metaplasia involving the gastric corpus (31).

The replacement of atrophic gastritis by intestinal metaplasia in the staging of gastritis considerably increased interobserver agreement, with the correlation with the severity of gastritis remaining at least as strong (2, 29). OLGIM should be preferred over the OLGA for predicting of gastric cancer risk in patients with premalignant lesions (2).

High OLGA and OLGIM stages have been found to be independent risk factors for gastric cancer, and may be useful for risk assessment in high-risk regions, especially for intestinal-type gastric cancer (12, 32). IM at a single location has a higher risk of gastric cancer. However, this increased risk does not justify surveillance in most cases, particularly if a high-quality endoscopy with biopsies has excluded advanced stages of atrophic gastritis (2). Advanced stages of atrophic gastritis and those with a family history of gastric cancer may benefit from a more intensive follow-up (e.g., every 1–2 years after diagnosis) (2).

Patients with advanced stages of atrophic gastritis (severe atrophic changes or intestinal metaplasia in both antrum and corpus, OLGA/OLGIM III/IV, EGGIM scores 5–10) have increased the risk of gastric cancer and should be followed up with a high quality endoscopy every 3 years (2, 24, 26, 30).

The Kyoto classification score in patients without a history of *H. pylori* eradication of 0, 1, and ≥2 was found to be associated with *H. pylori* infection rates of 1.5, 45, and 82%, respectively (24). Kyoto classification scores of ≥4 may be associated with increased gastric cancer risk (24). A modified Kyoto classification, which included open-type endoscopic atrophy, invisible regular arrangement of collecting venules at the incisura, virtual CE detecting intestinal metaplasia in >30% of the corpus and map-like redness in the corpus, performs better in determining EGC risk than the original Kyoto classification (30).

## Detection and Characterization of Early Gastric Cancer

An adequate endoscopic evaluation with a histological diagnosis of EGC is crucial in order to plan an endoscopic therapeutic strategy (1). Endoscopic features of a suspicious lesion for EGC include focal erythema or pallor, irregularity of the mucosal surface (protrusions, elevated or depressed lesions), altered mucosal folds, and spontaneous bleeding (19). Conventional white light endoscopy is useful to evaluate for ulcers and ulcer scars in EGC as well as the depth of submucosal invasion with convergence of folds and tenting at the site of EGC, suggesting deep submucosal invasion (1, 19, 33). IEE technology with FICE with pCLE guided targeted biopsies has been shown to more than double the diagnostic yield of gastric intestinal metaplasia, dysplasia, and early gastric cancer while decreasing the number of biopsies by up to 50% when compared to FICE and random biopsies (8).

In cases of doubt regarding the depth of submucosal invasion, echoendoscopic evaluation can be useful in characterization of the depth of submucosal invasion as well as ruling out loco regional lymph node metastases (1, 33).

**Figure 2:**
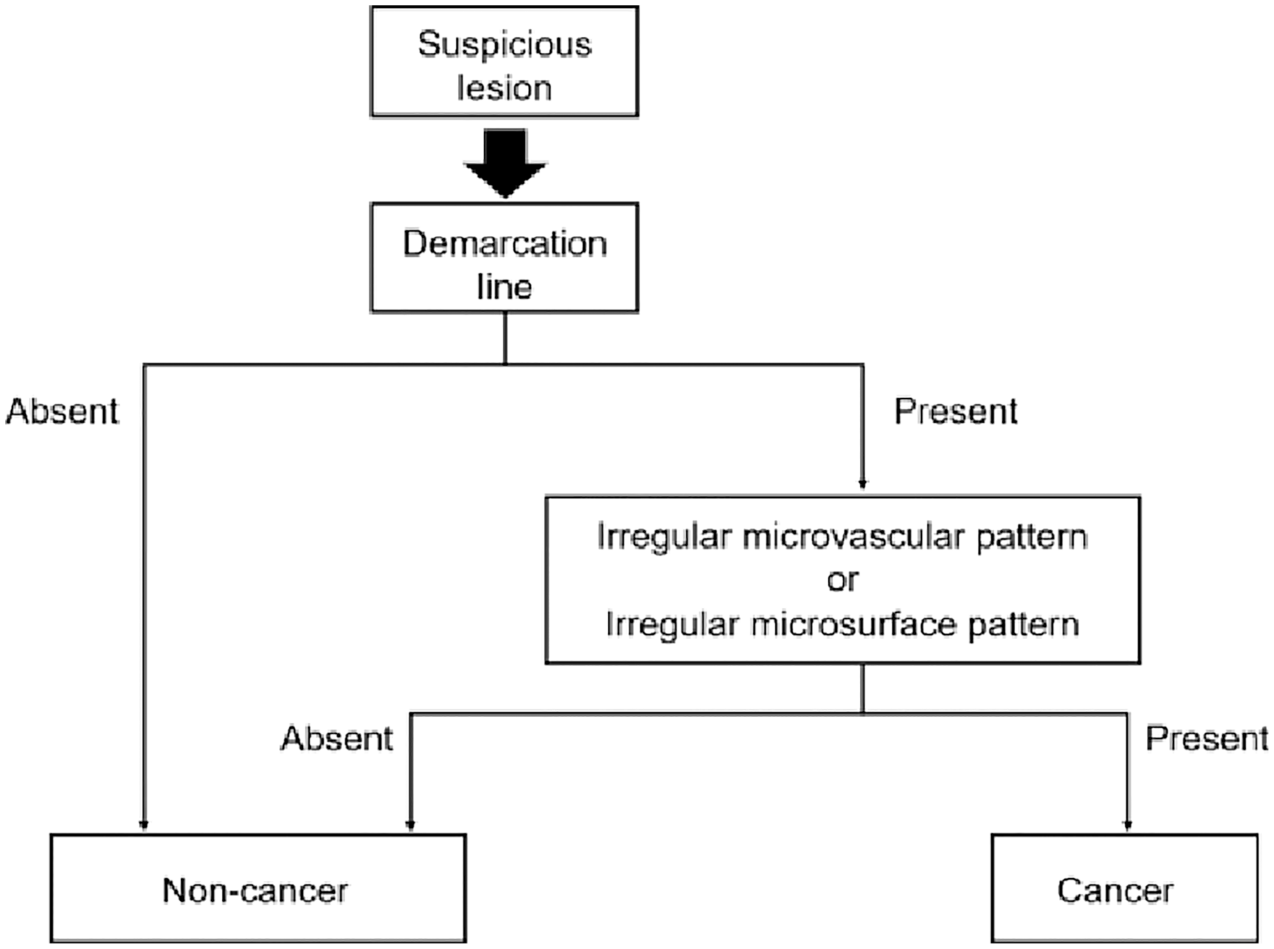
Diagnostic algorithm for gastric cancer with magnifying endoscopy. Reproduced with permission from Yao K et al. (1)

**Figure 3:**
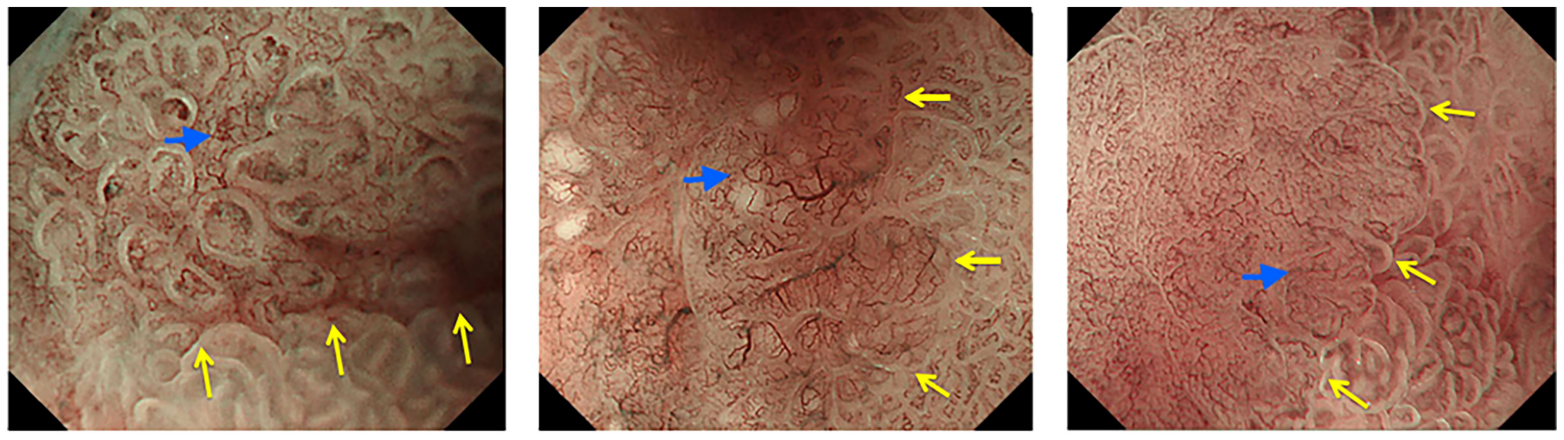
Demarcation line (yellow arrows) and irregular microvascular pattern (blue arrows) suggesting early gastric cancer. Reproduced with permission from Muto M et al. (19)

The Japanese Gastroenterological Endoscopy Society has proposed a magnified endoscopy simple diagnostic algorithm (MESDA) for gastric cancer (**Figure 2**) (1, 19). The microvascular pattern (MV) is comprised of a subepithelial capillary (SEC), a collecting venule (CV), and pathological microvessels (MVs) while the microsurface (MS) pattern is identified by marginal crypt epithelium (MCE), crypt opening (CO) and an intervening part (IP) between crypts (1). The demarcation line is a border between the lesion and non-lesion areas which is perceptible through an abrupt change in MV and or MS patterns. In the presence of subtle mucosal changes such as redness or polypoid or depressed lesions, the absence of a demarcation suggests a non-cancer. If a demarcation line is present, an irregular MS pattern or an irregular MV pattern suggest the presence of cancer as shown in **Figure 3** (1, 19, 23). Demarcation line determination may be difficult in undifferentiated EGC and in certain differentiated EGCs, requiring biopsies from the surroundings of the lesion (33). Overall, MESDA for EGC has a pooled sensitivity of 83%, specificity of 89%, and a high diagnostic accuracy of around 95% with a positive predictive value of 79% and a negative predictive value of 99% (19, 25).

Patients with dysplasia detected in the gastric mucosa should be referred to a reference center, with all dysplastic lesions resected and in the absence of an endoscopically visible lesion, repeat endoscopy performed within 6 months if high-grade dysplasia and within 6 to 12 months if low-grade dysplasia (25). After the resection of EGC, patients should undergo yearly endoscopic surveillance to detect metachronous EGC (25).

Finally, a gastric cancer detected within 3 years of upper gastrointestinal endoscopy is considered a failure to detect cancer and should be auditable (15). Missed gastric cancers after upper gastrointestinal endoscopy vary between 4.6 and 14.4% and should be less than 10% in an endoscopy unit (15, 25).

In conclusion, adequate preparation of the upper digestive tract, risk stratification, and careful inspection of the gastric mucosa with high definition endoscopes with image enhanced endoscopic technology is crucial for the detection of early gastric cancer.

## Author Contributions

CNF prepared the manuscript and revised it for intellectual content. JS provided intellectual input for the manuscript and prepared part of the manuscript and revised it for intellectual content. RM revised the manuscript for intellectual content. All authors listed have made a substantial, direct, and intellectual contribution to the work and approved it for publication.

## Conflict of Interest

The authors declare that the research was conducted in the absence of any commercial or financial relationships that could be construed as a potential conflict of interest.

## Publisher’s Note

All claims expressed in this article are solely those of the authors and do not necessarily represent those of their affiliated organizations, or those of the publisher, the editors and the reviewers. Any product that may be evaluated in this article, or claim that may be made by its manufacturer, is not guaranteed or endorsed by the publisher.
